# Incomplete phenotypic presentation in a girl with rare Rabson–Mendenhall syndrome

**DOI:** 10.1007/s00592-022-01971-3

**Published:** 2022-11-04

**Authors:** Joanna Chrzanowska, Jagoda Skarul, Agnieszka Zubkiewicz-Kucharska, Maciej Borowiec, Agnieszka Zmyslowska

**Affiliations:** 1 Department of Endocrinology and Diabetology for Children and Adolescents, Wroclaw Medical University, Wrocław, Poland; 2 Department of Clinical Genetics, Medical University of Lodz, Pomorska Str. 251, 92-213 Lodz, Poland

**Keywords:** Insulin resistance, Diabetes mellitus, Rabson–Mendenhall syndrome, Genetics

## Introduction

Severe insulin resistance syndromes are rare genetic disorders divided into: lipodystrophies, primary insulin signalling defects and complex insulin resistance syndromes. In contrast to lipodystrophies, severe insulin resistance due to insulin receptoropathy is associated with the absence of non-alcoholic steatohepatitis, a normal lipid profile, normal or high levels of adiponectin and sex hormone-binding globulin (SHBG). There are three main types of insulin receptoropathy related to the characteristic clinical features and type of causative variant found in the insulin receptor gene (*INSR*): type A insulin resistance (TAIR), Rabson–Mendenhall syndrome (RMS) and Donohue syndrome (DS). Hypoglycaemia/hyperglycaemia is present in each of the above diseases, with a risk of developing insulin-resistant monogenic diabetes due to an insufficient compensatory response of the pancreatic beta cells to regulate glucose metabolism [1].

Patients with RMS (OMIM: 262190, ORPHA 769) are usually homozygotes or compound heterozygotes for mutations affecting the alpha subunit of INSR and have the following symptoms in addition to *acanthosis nigricans*: growth abnormalities, abnormal teeth, pineal hyperplasia and facial dysmorphia (such as abnormally large ears, full lips, and a furrowed tongue). There is no established and effective causal treatment for RMS syndrome [1].

The case of a girl with severe insulin resistance since early childhood and additional features is presented below.

## Clinical presentation

We present the case of an 11-year-old girl, daughter to non-consanguineous parents born by caesarean section at 40 weeks’ gestation, with normal birth weight (3100 g, standard deviation score (SDS) = − 1.17), in good condition. Her mother had a family history of insulin resistance (HOMA-IR index: 9.81, without hyperglycaemia but with fasting hyperinsulinaemia). The girl's father was diagnosed with hypoglycaemia with normal fasting insulin levels.

**Fig. 1:**
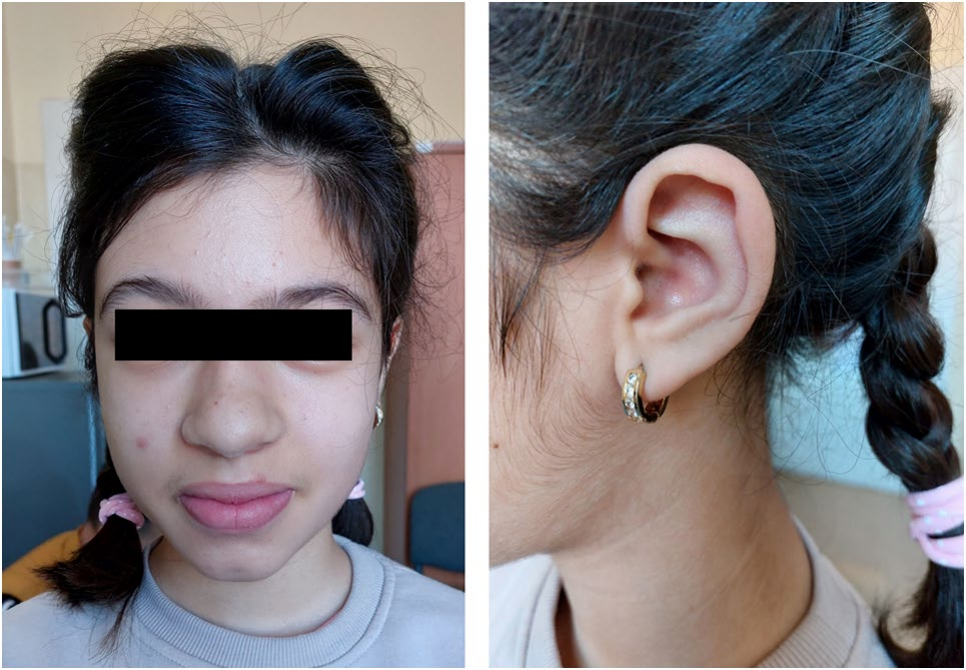
Features of facial dysmorphia present in the patient

The patient was admitted to the Department of Pediatric Endocrinology and Diabetology at the age of 9 years because of hypertrichosis (since birth) and dark discolouration in the body folds (noticed a few months before the first hospitalisation). On admission, physical examination of the girl revealed: overweight, *acanthosis nigricans* localized in armpits, groin and back neck and hypertrichosis, particularly severe on the back and limbs. The patient presented facial dysmorphic features such as thick lips, wide nasal root, bulbous nasal tip, hypertelorism, large, low set ears (Fig. 1) and high-arched palate. Pubertal status was assessed at stage two on the Tanner scale. The auxological examination reported: height (Ht) 130 cm (HtSDS = − 0.79) and body mass index (BMI) 19.9 kg/m^2^ (BMI SDS = 1.4) (Table 1). Laboratory results showed significant fasting hyperinsulinaemia with glucose levels at the lower limit of normal (61 mg/dL). The HOMA-IR index was above 45. The OGTT result mandated the diagnosis of impaired glucose tolerance and hyperinsulinaemia (Table 2). The HbA1c level was 5.4%. Despite severe insulin resistance, liver function and fasting lipid profile were normal. SHBG was elevated by 172 nmol/L, while androgens were within normal range. Pelvic ultrasonography showed ovarian enlargement without polycystic changes (Table 1). Body composition analysis by bioimpedance showed an increased percentage of body fat in the total body weight (32%). Dietary modifications did not affect insulin secretion. Further examination showed normal leptin levels (10.48 ng/mL), but high adiponectin levels (31.07 ug/mL). IGF-1 and IGFBP-3 concentrations were within the normal range (160 ng/mL and 4.76 ug/mL, respectively).

**Table 1:** Laboratory tests result in a patient within a follow-up

Parameter at the age of: (years)	8.9	9.2	10.6 (implementation of metformin therapy)	11.1	11.4
BMI (body mass index) (kg/m^2^)	19.9	19.3	17.45	17.45	17.8
SDS (standard deviation score)-BMI	1.4	1.2	-0.1	-0.1	-0.01
HbA1c (%/mmol/mol)	5.4/36	5.4/36	7.6/60	6/42	5.1/32
fasting glucose (mg/dL)	61/46	53	44	57	47/60
Normal range 59–99					
fasting insulin level (μIU/mL)	> 300/58.8	196	300	295	46.1/272
Normal range < 29					
HOMA-IR (homeostatic model assessment insulin resistance) index	> 45.1/6.7	25.6	32.5	41.5	5.3/40.3
ASPAT/ALAT(U/L)	27/21	30/21	25/16	19/14	20/10
Normal range 0–45/0–40					
Total cholesterol (mg/dL)	174	167	189	177	125
Normal range < 190					
LDL cholesterol (mg/dL)	77	70	N/A	79	47
Normal range < 135					
HDL cholesterol (mg/dL)	86	83	N/A	87	72
Normal range > 40					
Triglyceride (mg/dL)	54	69	N/A	57	31
Normal range < 150					
Androstendione (ng/mL)	0.748	0.502	4.64	6.64	4.38
Normal range 0.3–3.3					
Testosterone (ng/mL)	< 0.2	< 0.2	1.35	2.74	1.2
				Normal range < 0.8	Normal range < 0.8
SHBG (sex hormone-binding globulin) (nmol/L)	172	164	159	N/A	N/A
Normal range 18–144					
FAI (free androgen index)	0.4	N/A	2.9	5.95	N/A
Normal range < 5					
Volume of the ovaries (mL)	4.2 and 4.6			13.1 and 11.4	8.5 and 9.1
	Normal range 1.2–2.3			Normal range 2–4	Normal range 2–4

**Table 2:** The results of the OGTT tests performed during follow-up

	Time (minutes)	0’	60’	120’	180’
At the age of 8.9	Glucose (mg/dL)	61	162	183	N/A
	Insulin (μIU/mL)	> 300	> 300	> 300	N/A
At the age of 9.2	Glucose (mg/dL)	53	119	150	126
	Insulin (μIU/mL)	196	897	1400	1840
At the age of 11.1	Glucose (mg/dL)	57	225	191	87
	Insulin (μIU/mL)	295	1216	218	1143
At the age of 11.4 (on metformin therapy)	Glucose (mg/dL)	60	146	168	193
	Insulin (μIU/mL)	272	486	920	1010

During follow-up hospitalizations, the child's clinical status was assessed, and glycaemia and insulinaemia were monitored during OGTT testing (Table 2). Other laboratory tests were also carried out, as shown in Table 1. Despite a reduction in BMI, an increase in hypertrichosis and *acanthosis nigricans* was still observed.

**Fig. 2:**
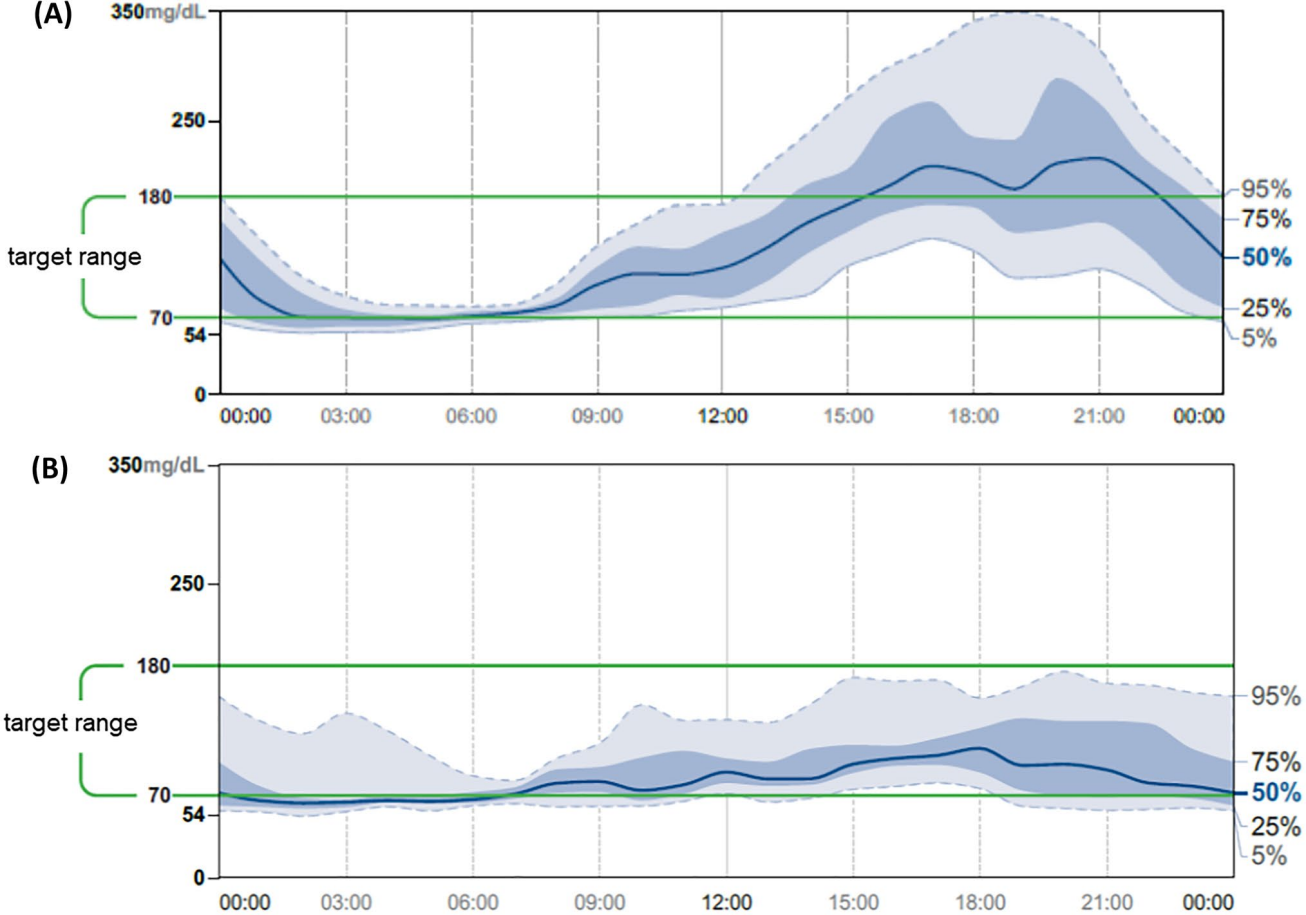
Results of flash glucose monitoring (FGM) in a patient: A. at diagnosis of diabetes; B. during treatment with metformin

At the age of 10 and a half years, the girl was diagnosed with diabetes, based on HbA1c 7.6%. No clinical signs of diabetes were observed. C-peptide level was normal, and diabetes-related autoantibodies were negative. At this time, the patient was monitored using flash glucose monitoring (FGM), as shown in Fig. 2. The daily glycaemic pattern showed low glucose levels at night and in the morning and high glucose levels in the evening for the first two days (Fig. 2A). On the third day of FGM, treatment with metformin was started at an initial dose of 500 mg once a day and gradually increased to a dose of 1000 mg (500 mg twice a day). After 6 months of metformin treatment, an HbA1c reduction of 6% was achieved. The metformin dose was increased to the target dose of 2000 mg daily. At this time, an increase in the degree of hyperandrogenemia was also noticed (Table 1). The ovaries were enlarged and polycystic. Bone age was accelerated by 2 years.

A follow-up physical examination revealed significant facial dysmorphia and slight clitoral enlargement, as well as dental malocclusion and crowding of the teeth. The patient was prone to dental caries and remains under the care of an orthodontist and dentist. She was not menstruating. The auxological examination showed Ht 148 cm (HtSDS = − 0.24) and BMI 17.8 kg/m^2^. HbA1c level decreased to 5.1%. The FGM result (during metformin treatment) showed a mean glucose level of 90 mg/dL, and the percentage of time the glucose level was within the range of 54–180 mg/dL was 98% (Fig. 2B). After 10 months of metformin treatment, androgens were above normal but tended to decrease. Interestingly, during the whole of the patient's medical follow-up, IGF-1 values remained normal. Pelvic ultrasonography confirmed that the ovaries were enlarged (Table 1). Abdominal ultrasonography showed no organomegaly and excluded nephrocalcinosis. The echocardiographic result was normal. Brain MRI showed a normal pineal gland volume with a cyst, which, however, was not clinically significant. A dual-energy X-ray absorptiometry study showed a total body fat content of 28.4% (normal range 15–25%).

Due to suspected insulin receptoropathy, the patient was referred to the Rare Diseases of Children and Adolescents Outpatient Clinic of the Department of Clinical Genetics for genetic testing.

## Molecular analysis

In the Department of Clinical Genetics of the Medical University of Lodz, Poland, the patient underwent next-generation sequencing (NGS) analysis including coding sequences and adjacent intron sequences of the *INSR* gene which confirmed the presence of 2 variants.

The first was found to be a heterozygous variant c.3720_3723delGTCT located in exon 21, leading to the conversion of serine to methionine at position 1241 of the polypeptide chain, a shift in the reading frame and premature termination of the polypeptide chain. The identified variant was described in the ClinVar, dbSNP and HGMD databases and classified according to the American College of Medical Genetics and Genomics (ACMG) guidelines as pathogenic.

The second was a heterozygous c.3038C > T variant located in exon 17, leading to the conversion of proline to leucine at position 1013 of the polypeptide chain, which was described in the dbSNP database, but not in the ClinVar and HGMD databases. In silico substitution analysis of p. Pro1013Leu using the prediction programmes MutationTaster and PROVEAN indicated its damaging nature. The latter variant is classified according to ACMG recommendations as VUS (variant of uncertain significance), but when it appears de novo or segregates with disease symptoms, it is classified as probably pathogenic. In order to analyse the segregation of mutations in the patient's family, a molecular test was also performed in her parents, finding the presence of the heterozygous pathogenic variant c.3720_3723delGTCT of the *INSR* gene in the patient's mother and the presence of the heterozygous variant c.3038C > T of the *INSR* gene in the patient's father. Finally, the second variant was found to be probably pathogenic.

Thus, taking into account the whole clinical picture and the results of molecular, laboratory and imaging studies, the diagnosis of RMS should be considered as the most appropriate one.

## Discussion

This case report presents the diagnostic difficulties associated with the diagnosis of RMS syndrome. This description presents a patient with the presence of two causative variants in the *INSR* gene and typical phenotypic features of RMS, but without short stature and with normal IGF-1 levels. To date, only isolated cases of RMS have been described in which, at least at some stage of postnatal life, body height was within normal limits (without IGF-1 therapy) [2]. Low IGF-1 levels therefore seem to be commonly observed in patients with RMS/DS [1, 2]. Only Dutta et al. described a girl suspected of having RMS (the diagnosis was made on the basis of clinical presentation without genetic testing), whose IGF-1 was in the lower limits of normal [3]. The exact cause of impaired growth hormone (GH) secretion in RMS is not clear. Since insulin signalling is impaired in RMS, it appears that insulin in high concentrations may inhibit GH secretion through cross-talk signalling via the IGF-I receptor. There is also evidence to suggest that insulin increases hepatic IGF-1 and IGFBP-3 production through direct regulation of the GH receptor, and thus, in patients with severe insulin resistance, IGF-1 deficiency may be due to both impaired hepatic production and accelerated IGF-I excretion [1]. Individuals with RMS are therefore typically small relative to gestational age and exhibit postnatal short stature of varying severity [4, 5].

In most of them, symptoms appear already in the first year of life [4], but sometimes characteristic clinical features are present later, at the age of 2–11 years [3, 5]. Our patient was born with a normal body weight and developed normally also after birth, but she showed features of facial dysmorphia and abnormalities of the masticatory organ. The first signs of insulin resistance in the form of *acanthosis nigricans* appeared when the girl was about 8 years old, which was the reason for starting the medical diagnosis, which consequently led to the diagnosis of hyperglycaemia with severe insulin resistance.

Thus, the symptoms of RMS vary between individuals, and affected individuals do not have all symptoms. Nephrocalcinosis, renal enlargement or medullary sponge kidney have also been described as features of RMS [5], but were not observed in our patient. Also, establishing a genotype–phenotype correlation for the *INSR* gene variants seems to be difficult. Pathogenic mutations can alter INSR function through various mechanisms, such as reduced expression of the receptor on the cell surface, impaired insulin binding and/or autophosphorylation of the receptor or impaired recycling kinetics. The more severe nature of the symptoms in patients with RMS is probably due to the defect in both alleles of the *INSR* gene, especially the alpha subunit [2]. Takeuchi et al. noted that homozygous or compound heterozygous causative mutations were found in 87.5% of RMS/DS patients, with significant variation in their location within the *INSR* gene [4].

Another challenge seems to be to undertake effective treatment in patients with RMS, while the causal one is not defined. It has been shown that infusion of IGF-1 or leptin may be a promising therapeutic option for patients with insulin receptoropathy. However, IGF-1 therapy was able to improve metabolic control of diabetes and growth in some of these described cases but remained ineffective in others [1].

In our patient, large diurnal fluctuations in insulin levels and mainly fasting hypoglycaemia were observed, but without the clinical signs typical for hypoglycaemia. In insulin receptoropathy syndrome, impairment of glucose metabolism includes both hypoglycaemia and hyperglycaemia. The duration of the disease influences the nature of the carbohydrate disturbances. Initially, episodes of alternating hypoglycaemia and hyperglycaemia predominate, and eventually, a progressive decline in insulin levels leads to insulin-resistant diabetes [1]. However, fasting hypoglycaemia is a hallmark of RMS. In fact, less efficient hepatic fasting glucose production, due to impaired hepatic glycogen synthesis during feeding, leads to fasting hypoglycaemia and postprandial hyperglycaemia. The deficiency or the absence of adipose tissue observed in some patients with RMS is the result of impaired both insulin-induced triglyceride synthesis and adipocyte differentiation [4].

Our patient was initially overweight, which was no longer observed as a result of lifestyle modifications, which, combined with metformin therapy, effectively improved metabolic control of diabetes.

## Conclusion

The variability of the clinical presentation of congenital insulin receptoropathy should be emphasized. Thus, the absence of low stature with normal IGF-1 values throughout the follow-up period does not exclude the diagnosis of RMS.
